# Investigating the effects of copy number variants on reading and language performance

**DOI:** 10.1186/s11689-016-9147-8

**Published:** 2016-05-15

**Authors:** Alessandro Gialluisi, Alessia Visconti, Erik G. Willcutt, Shelley D. Smith, Bruce F. Pennington, Mario Falchi, John C. DeFries, Richard K. Olson, Clyde Francks, Simon E. Fisher

**Affiliations:** Language and Genetics Department, Max Planck Institute for Psycholinguistics, Wundtlaan 1, 6525 XD Nijmegen, The Netherlands; Department of Translational Research in Psychiatry, Max Planck Institute of Psychiatry, Munich, Germany; Department of Twin Research and Genetic Epidemiology, King’s College London, London, UK; Institute for Behavioral Genetics, University of Colorado, Boulder, CO USA; Department of Psychology and Neuroscience, University of Colorado, Boulder, CO USA; Munroe Meyer Institute, University of Nebraska Medical Center, Omaha, NE USA; Department of Psychology, University of Denver, Denver, CO USA; Donders Institute for Brain Cognition and Behaviour, Nijmegen, The Netherlands

**Keywords:** Reading disability, Developmental dyslexia, Language, Reading, Copy number variants, Family-based GWAS, Meta-analysis, CLDRC

## Abstract

**Background:**

Reading and language skills have overlapping genetic bases, most of which are still unknown. Part of the missing heritability may be caused by copy number variants (CNVs).

**Methods:**

In a dataset of children recruited for a history of reading disability (RD, also known as dyslexia) or attention deficit hyperactivity disorder (ADHD) and their siblings, we investigated the effects of CNVs on reading and language performance. First, we called CNVs with PennCNV using signal intensity data from Illumina OmniExpress arrays (~723,000 probes). Then, we computed the correlation between measures of CNV genomic burden and the first principal component (PC) score derived from several continuous reading and language traits, both before and after adjustment for performance IQ. Finally, we screened the genome, probe-by-probe, for association with the PC scores, through two complementary analyses: we tested a binary CNV state assigned for the location of each probe (i.e., CNV+ or CNV−), and we analyzed continuous probe intensity data using FamCNV.

**Results:**

No significant correlation was found between measures of CNV burden and PC scores, and no genome-wide significant associations were detected in probe-by-probe screening. Nominally significant associations were detected (*p*~10^−2^–10^−3^) within *CNTN4* (contactin 4) and *CTNNA3* (catenin alpha 3). These genes encode cell adhesion molecules with a likely role in neuronal development, and they have been previously implicated in autism and other neurodevelopmental disorders. A further, targeted assessment of candidate CNV regions revealed associations with the PC score (*p*~0.026–0.045) within *CHRNA7* (cholinergic nicotinic receptor alpha 7), which encodes a ligand-gated ion channel and has also been implicated in neurodevelopmental conditions and language impairment. FamCNV analysis detected a region of association (*p*~10^−2^–10^−4^) within a frequent deletion ~6 kb downstream of *ZNF737* (zinc finger protein 737, uncharacterized protein), which was also observed in the association analysis using CNV calls.

**Conclusions:**

These data suggest that CNVs do not underlie a substantial proportion of variance in reading and language skills. Analysis of additional, larger datasets is warranted to further assess the potential effects that we found and to increase the power to detect CNV effects on reading and language.

**Electronic supplementary material:**

The online version of this article (doi:10.1186/s11689-016-9147-8) contains supplementary material, which is available to authorized users.

## Background

Reading disability (RD or developmental dyslexia) and specific language impairment (SLI) are two of the most prevalent neurodevelopmental disorders, with a prevalence of ≈5–8 % among school-aged children (as reviewed in [1–3]). Both RD and SLI are multifactorial disorders with moderate to high heritabilities and are characterized by high comorbidity, also with other neurodevelopmental disorders such as attention deficit hyperactivity disorder (ADHD) and speech sound disorders (SSD) [2, 4, 5]. It is likely that RD and SLI, as well as the underlying reading- and language-related skills, share some genetic/neurobiological mechanisms [6, 7].

Candidate genes that have been implicated in reading- and language-related traits include *DYX1C1* (15q21), *KIAA0319* and *DCDC2* (6p22), *MRPL19/GCFC2* (2p12), *ROBO1* (3p12), *CNTNAP2* (7q35), *CMIP* and *ATP2C2* (16q23-24), and *FOXP2* (7q31) (see [8–10] for reviews). More recently, genome-wide association scans (GWAS) using measures of both reading and language have reported suggestive associations in *ABCC13* (21q11.2), *DAZAP1* (19p13.3), *ZNF385D* (3p24.3), *FLNC* (7q32.1), and *RBFOX2* (as reviewed in [10]). Several of these candidate genes are known to have roles in important processes in central nervous system (CNS) development, such as neuronal migration, axonal guidance, and neurite outgrowth [8]. Moreover, a link with steroid hormone-related biology has also been hypothesized (see [11] for further details).

In these genes, most of the variants that have been tentatively associated with reading and/or language traits are single-nucleotide polymorphisms (SNPs), although other types of genetic variants have also been implicated. These include balanced translocations disrupting *ROBO1* [12] and *DYX1C1* [13] in dyslexic cases and translocations and deletions affecting *FOXP2* in a severe form of speech and language disorder, involving childhood apraxia of speech (CAS) [14].

The putative genetic associations reported so far can explain only a small proportion of heritable variance in reading and language skills [1, 4, 10]. Part of the “missing heritability” may be represented by Copy Number Variants, defined as structural variations in the genome that result in regions larger than 1 kb showing a non-diploid copy number. Several copy number variants (CNVs) have been identified in severe neurodevelopmental and neuropsychiatric disorders, including schizophrenia (SCZ), Autism Spectrum Disorders (ASD), Intellectual Disability (ID) and Developmental Delay (DD) [15, 16]. However, only a few studies have focused on reading and/or language impairments, which we review briefly here. In the majority of these studies, a perfect co-segregation between CNVs and poor reading/language performance has seldom been observed.

In a recent investigation on ten Indian dyslexic families, de novo CNVs were identified at several loci, namely *GABARAP* (17p13.1), *NEGR1* (1p31.1), *ACCN1* (17q11.21), *DCDC5* (11p14.1), and the known SLI candidate gene *CNTNAP2* (7q35) [17]. In the same families, candidate susceptibility CNVs affecting the *PCDH11X* gene (Xq21.31-q21.32) were also identified [18]. In a Dutch family, Poelmans and colleagues [19] identified a heterozygous deletion in 21q22.3 co-segregating with RD and encompassing the genes *PCNT*, *DIP2A*, *S100B*, and *PRMT2*.

The largest study to date on CNVs in dyslexia involved 376 RD cases and 337 controls. Candidate susceptibility CNVs were found, overlapping *IMMP2L* and *AUTS2* (7q11.22) [20], a well-known ASD susceptibility locus.

With regard to language impairments, Wisznieski et al. [21] identified a heterozygous deletion disrupting the gene *TM4SF20* (2q36), co-segregating with language delay in 15 Southeast Asian families. In a CNV scan of SLI families, a ~21-kb exonic microdeletion within *ZNF277* (7q31.1, adjacent to the *IMMP2L/DOCK4* locus) was found [22]. A genome-wide CNV study comparing 127 independent SLI cases from the same dataset, together with first-degree relatives and unrelated controls, reported novel candidate de novo CNVs [23], disrupting the genes *ACTR2* (2p14), *CSNK1A1* (5q33.1), and the regions typically involved in 22q11.2 and 8p23.1 duplication syndromes. A recent CNV screen in a longitudinal cohort of children with language-related difficulties or family risk of dyslexia revealed a de novo deletion in 15q13.1–13.3, observed in a child with persistent language impairment, normal reading skills, and no evidence of sensory or neurological problems [24]. This large heterozygous deletion had been previously reported in cases of broader neurodevelopmental delay [24].

Other CNVs have been associated with poor reading or language performance in the context of other comorbid disorders. A deletion disrupting both *DOCK4* and *IMMP2L* (7q31.1) was found to co-segregate with poor reading performance in a family with two ASD cases, and another *DOCK4* exonic deletion co-segregated with RD in a distinct dyslexic family [25]. Canonical 16p11.2 microdeletions—usually implicated in mild cognitive impairment, general developmental delay, speech and language problems, and ASD—have been associated with CAS by independent studies [26, 27]. The same microdeletion was hypothesized to act jointly with a 6q22.31 duplication in a subject with CAS and pervasive developmental disorder [28]. Prader-Willi/Angelman patients, presenting deletions/duplications of the 15q11.2 region, have been reported to frequently exhibit speech and language delays [29]. Similarly, subjects with 2p15-p16.1 microdeletion syndrome typically show cognitive, linguistic, and psychiatric disabilities. In this region, a de novo deletion encompassing *BCL11A* has been implicated in a mild phenotype characterized by apraxia, dysarthria, and expressive language delay [30].

Recently, Stefansson and colleagues [31] investigated the effect of several CNVs previously associated with SCZ or ASD (hereafter called “neuropsychiatric CNVs”) on different cognitive traits in a large Icelandic sample (*N*~102,000). By comparing SCZ patients, neuropsychiatric CNV carriers, other CNV carriers, and general population controls, they found that neuropsychiatric CNV carriers performed at a level between SCZ patients and controls on several psychometric tests, suggesting an effect of these CNVs on general cognition. Some neuropsychiatric CNVs showed association with cognitive abilities: among these, 16p11.2del and 22q11.21dup were associated with category and letter fluency, while 15q11.2del was associated with a history of dyslexia and dyscalculia.

In the present study, we have further investigated the potential influence of CNVs on reading and language performance through a comprehensive set of analyses, including total genome-wide CNV burden testing and two complementary methods to screen the genome for individual CNVs that may affect these traits. We used a dataset that has been previously included in a SNP-based GWAS meta-analysis (GWASMA) of reading and language traits [11], composed of children recruited for school history of RD or ADHD, and their unaffected siblings.

Current CNV research in psychiatric genetics often relies on case/control dichotomous classifications and seldom detects perfect co-segregation between CNVs and disease status. When heritable quantitative traits are available that are strongly correlated with a dichotomous definition of a disorder—as in the case of reading/language traits—analyzing the effect of putative CNVs directly on the quantitative trait provides an effective alternative to the analysis of co-segregation between CNVs and the disorder. The former analysis is aimed at detecting variants with reduced penetrance and variable expressivity on traits of interest, while the latter one is aimed at detecting variants with full penetrance and expressivity. We used both approaches in our study.

## Methods

**Fig. 1:**
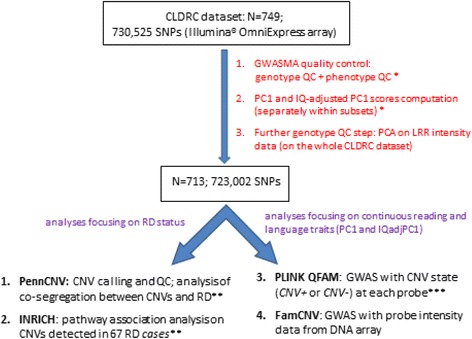
Experimental workflow and dataset analyzed in the present study. (*Single asterisk*) As described in [11]. (*Double asterisks*) RD cases were defined as samples in the lowest 10 % of IBGdiscr score distribution. (*Triple asterisks*) Legend of CNV states: “*CNV+*” corresponds to copyN ≠ 2 (≠1 for X chromosome probes in males); “*CNV−*” corresponds to copyN = 2 (=1 for X chromosome probes in males). See “GWAS with CNV state” section for further details

The experimental workflow of the present study, described in this section, is summarized in Fig. 1. For simplicity, genotype and phenotype quality control (QC) are described below in single paragraphs.

### Dataset

The dataset analyzed in the present work was collected in the Colorado Learning Disabilities Research Centre (CLDRC) study, an ongoing research project on the etiology of learning disabilities carried out in 27 school districts in Colorado, USA [32]. Briefly, pairs of twins were recruited for a school history of RD or ADHD in at least one of the twins; they were then administered a number of psychometric tests for several learning-related skills, along with their additional co-siblings, and DNA was collected for genetic studies. The Institutional Review Boards of the University of Nebraska Medical Center and of the University of Colorado at Boulder had approved the protocol, and written informed consent of the participants (or their parents) was obtained.

For MZ twin pairs, we selected one child per pair based on the maximum availability of reading- and language-related trait data or otherwise randomly. The sample of twins and siblings available for this study comprised 749 participants in total (mean age 11.7 years, age range 8–19), from 343 unrelated twinships/sibships. Of these, 266 of the twinships/sibships (a total of 585 participants) were originally recruited via a proband with a history of RD and 77 of the twinships/sibships (164 participants in total) were originally recruited via a proband with a history of ADHD. The two subsets are indicated hereafter as CLDRC-RD and CLDRC-ADHD.

### Reading and language measures

The reading- and language-related traits assessed in CLDRC are reported in Table 1, and the relevant measures are described in detail elsewhere [11]. These traits had been previously age-adjusted according to normative data and further rank-normalized when a measure differed significantly from normality. Phenotypic outliers were removed from the dataset, along with subjects with full-scale IQ <70 (two participants in CLDRC-RD in total). This left 564 subjects in CLDRC-RD and 163 in CLDRC-ADHD. Then, samples underwent separate principal component analyses (PCAs) in CLDRC-RD and in CLDRC-ADHD for the computation of the first principal component scores within each dataset, as briefly described below (further details in [11]).

**Table 1:** Phenotypic traits available and measures used for PC1 score derivation (labeled with relative loadings on PC1)

Trait	Description (ability assessed)	CLDRC-RD (564)	CLDRC-ADHD (163)
WRead	Reading real words	0.918	0.871
WSpell	Spelling real words	0.813	0.764
PD	Ability to convert letter strings into sounds, according to given phonetic rules	0.895, 0.861^a^	0.821, 0.729^a^
PA	Ability to recognize and manipulate speech sounds (phonemes)	0.801	0.744
OC	Ability to recognize a word as an orthographic unit and to retrieve the corresponding phonological form	0.764	0.644
NWR	Ability to repeat nonsense words orally presented	0.493	0.355
VIQ	Verbal reasoning		
PIQ	Logical reasoning		
PC1	Shared variance in reading and language skills	544 (528)	159 (155)
IQ-adjusted PC1	Shared variance in reading and language skills, not shared with performance IQ	544 (525)	159 (155)

Sample sizes of the datasets that underwent the PCA are reported in the header row. The number of samples for which PC1 score was computed are reported at the bottom of the table (as we excluded participants with at least one missing measure among the traits involved in the PCA). These numbers still include LRR outliers and samples discarded in CNV calling and QC process, which were filtered out for the specific purpose of this study, after extraction of PC1 scores. Final sample sizes at the end of all QCs are reported in brackets.

*WRead* word reading, *WSpell* word spelling, *PD* phonological decoding, *PA* phoneme awareness, *OC* orthographic coding, *NWR* nonword repetition, *VIQ/PIQ* verbal/performance IQ

^a^Loadings of nonword reading and phonological choice (respectively) on PC1s

#### First principal component score

The first principal component (PC1) from all of the language- and reading-related traits available (Table 1) was derived in each dataset, through the SPSS® 20.0 Factor Analysis. Only linear components with Eigenvalue >1 were extracted, allowing for correlation among the components (oblique rotation) and excluding subjects with any missing measure. PC1 explained 64.5 % of the total variance in CLDRC-RD and 52 % in CLDRC-ADHD, while PC2 explained no more than 13 % of the total variance in both datasets. PC1 scores showed a broad pattern of loadings across the traits in both datasets (Table 1). To obtain a measure of shared variance in reading and language skills independent of general cognitive abilities, we also regressed PC1 against performance IQ (which had not been included in PC1 computation), again separately within the two datasets, and used the residuals as IQ-adjusted PC1 scores (IQadjPC1).

#### IBG discriminant score

We also used an additional phenotypic trait, the IBG discriminant score (called IBGdiscr hereafter), a discriminant function empirically developed to diagnose RD in the context of the CLDRC study [33]. This score is a composite measure of word recognition, spelling, and reading comprehension tests (further details available in Additional file 1). For the purpose of the present study, we used IBGdiscr to select all the participants in the first and tenth decile of the score distribution (Additional file 1: Figure S1a), namely all the subjects with a standardized IBGdiscr <−1.4 (*N* = 67) and >2.2 (*N* = 69), as representative of poor and good reading performance, respectively. For simplicity, we indicate these subjects as “RD cases” and “controls” in the analyses where a dichotomous case-control classification was needed (see below).

Pairwise trait correlations of the reading and language composite/component scores analyzed—computed as median Pearson’s *r* coefficients over 100 repeat random samplings of one individual from each unrelated sibship—were high (*r*~0.83–0.98), both in CLDRC-RD and in CLDRC-ADHD (see Additional file 1: Table S1).

### DNA array data: generation and quality control

The two subsets were treated as a single dataset in DNA data generation and QC, as previously described in our GWAS meta-analysis [11]. DNA was extracted from whole blood or buccal swab samples and prepared for genotyping using standard protocols. DNA array data were generated using Illumina® Human OmniExpress array (730 k SNPs), and data were processed using Illumina’s GenomeStudio® software, following the manufacturer’s guidelines. QC and CNV calling process followed procedures already used in previous CNV studies [23, 34, 35]. Samples with genotyping success rate <95 % were discarded in GenomeStudio, along with probes mapping as “0” (no position) and “Y” (Y chromosome) and probes with call frequency <95 %. Using functions in the software PLINK v1.07 [36], we filtered out samples which showed inconsistencies in genome-wide identity-by-descent sharing with their siblings and unrelated individuals, or sex mismatches, or call rates <98 %, as well as homozygosity outliers, as described elsewhere [11].

As a further QC step for this study, we ran a PCA on the log R ratio (LRR) intensity signals of the 723,002 probes passing QC, through the *pca* command (*singular value decomposition* method) in the *pcaMethod* R package [37], extracting the first 100 principal components. This allowed us to assess the absence of extreme batch effects among the different plates of the microarray and to detect and remove 14 LRR outliers (Additional file 1: Figure S1d), which left 713 subjects for subsequent analysis.

### CNV calls

To detect CNVs, we applied PennCNV (version June 2011) [38] separately for autosomes and the X chromosome (704,855 and 18,147 SNPs, respectively), analyzing the two subsets jointly (*N* = 713). For this analysis, we built a custom population B allele frequency (PBF) file from our array intensity data through the *compile_pfb.pl* script in PennCNV, while default HMM parameters and GC model signal adjustment file were used. In order to obtain highly reliable CNV calls, we applied a series of filters to the CNV events initially called through the *detect_cnv.pl* script: only putative CNVs with a minimum confidence score of 10, covering at least 20 kb and 10 consecutive SNPs and showing limited overlap (<50 %) with Ig regions, pseudo-autosomal regions (PARs), centromeres, or other large genomic gaps were selected. Moreover, to ensure only high quality of samples, we filtered out samples showing an excessive number of CNV calls (>100 autosomal CNVs per sample) and LRR standard deviation >0.35. All the other parameters for sample filtering were set to default. Close CNVs were joined when the gap separating them was ≤20 % of the total length of the region that they covered. CNVs passing QC were finally annotated to RefSeq genes, within 50 kb beyond the 5′- and 3′-untranslated regions (UTRs), to include CNVs overlapping potential regulatory regions. Similarly, we annotated CNVs overlapping exons, and we identified a subset of “rare” CNVs, defined as CNV calls showing overlaps with less than five CNV events reported in the Database of Genomic Variants (DGV, July 2013 release, hg19). At the end of this process, we had 4490 final CNV calls for 702 samples, of which 3344 were annotated to genes, 2542 to exons, and 872 were rare.

#### Interpretation of CNVs and general statistics

The samples passing PennCNV QC (*N* = 702) were tested for correlation between their CNV burden—both in terms of total length and of total number of CNV events per sample—and our continuous traits of interest, namely PC1 and IQadjPC1, separately in the two subsets. This analysis was applied to 525 PC1/IQadjPC1 scores available in CLDRC-RD and to 155 scores available in CLDRC-ADHD. We repeated the same analysis on CNVs annotated to genes, on CNVs annotated to exons, and on rare CNVs (defined as above). Similarly, we analyzed correlations by length class, i.e., for short calls (<100 kb), medium calls (≥100 and ≤500 kb), and large calls (>500 kb) separately. To generate correlations unbiased by non-normality of CNV burden measures and by sample relatedness, rho correlation coefficients were calculated as the median rho over 100 repeat random samplings of one individual from each unrelated sibship, in R [39].

For the same classes of CNVs analyzed above (all, annotated and rare CNVs), we carried out a case-control burden analysis on 67 RD cases and 69 controls as defined above, through logistic regression of binary affection status versus CNV burden measures, over repeat random samplings of one individual from each unrelated sibship.

The final annotated CNVs were also assessed individually for co-segregation with the “RD case” status, focusing on large CNVs, on CNVs shared between two or more affected co-siblings, on CNVs affecting genes previously implicated in reading and language traits (see the “Background” section) or overlapping with other neuropsychiatric CNVs (previously tested by Stefansson et al. [31]).

### Genome-wide CNV association analyses of continuous reading and language PC traits

#### GWAS with CNV state

CNV calls made in PennCNV were also used for a genome-wide association test between CNV state at each probe and PC1/IQadjPC1. The alternative CNV states at each probe were “CNV-negative” (CNV−) when a probe showed a diploid copy number, and “CNV-positive” (CNV+) when it showed an abnormal copy number. In other words, both deletions and duplications at each probe were considered as a single CNV+ state.

We applied PLINK v1.07 QFAM analysis [36] to all the 43,525 probes covered by CNV events (41,625 autosomal probes and 1900 X chromosome probes), in CLDRC-RD (*N* = 525) and CLDRC-ADHD (*N* = 155) separately.

In order to have a bi-allelic coding for probes involved in this analysis, which indicated the presence or absence of a non-diploid state, proxy genotypes were created in the .ped input files. These were coded as “11” when the probes were not covered by any CNV (i.e., copy number =2) and as “12” when they fell within CNV calls (i.e., copy number ≠ 2). For chromosome X, CNV states per probe were coded as “11” for probes with copy number =1 and “12” for probes with copy number ≠1 in males, while they were coded following the rules of autosomal CNV state in females. Then, X chromosome probes were tested for association separately within males and females and later meta-analyzed. To correct for non-independence of siblings, permutations were run in QFAM analysis, as previously described in [11]. After QFAM analysis, the results of separate GWAS for CLDRC-RD and CLDRC-ADHD were meta-analyzed using the METAL software package, through the sample-size-based scheme [40]. Results were then interpreted in terms of consecutive probes showing significant associations (i.e., at least two consecutive probes with *p* < 0.005 at the genome-wide level and contiguous with two or more probes with *p* < 0.05), representing regions of overlap of two or more CNVs with potential effects on the continuous traits investigated.

#### GWAS with intensity data

As a complementary analysis, we tested for association of LRR and BAF (beta allele frequency) intensity data from our DNA array with PC1 and IQadjPC1, applying FamCNV 2.0 [41] (beta version available upon request to Dr. Mario Falchi).

In this analysis, we tested for association of 704,855 autosomal probes passing QC in CLDRC-RD (*N* = 525) and in CLDRC-ADHD (*N* = 155), using as covariates the first and second principal components computed in the PCA of LRR data (see the “DNA array data: generation and quality control” section). After running separate GWAS in the two subsets, the results were meta-analyzed as above, using rho correlation coefficients between LRR data and PC1/IQadjPC1 as beta values at each probe, indicative of the direction of association. Results were interpreted in terms of contiguous probes showing significant associations (i.e., pairs of consecutive probes with *p* < 0.001 and contiguous with two or more probes with *p* < 0.05), which were more likely to represent real CNV effects.

### Pathway-based analysis of CNV calls

To test specific molecular networks for enrichment of potentially disrupting CNVs, we ran a pathway-based association analysis in INRICH v1.0, through the *TARGET* algorithm [42]. This tool tests groups of independent genomic intervals for enrichment of overlaps with predefined gene sets, through a permutation-based approach. We initially tested 306 CNVs called in 67 RD cases, and then we restricted the analysis to 84 rare CNVs in the same subset.

Gene boundaries in the tested gene sets were again defined as extending 50 kb beyond the 5′- and 3′-UTRs, while random genomic intervals simulated in the permutations of the test were extracted from a reduced set of 43,525 SNPs, namely all the probes encompassed by CNV calls. We considered testing CNV calls more suitable than testing associated genomic intervals as produced by GWAS analyses, since such intervals would need to be defined on an LD basis, which is clearly inappropriate for the analysis of CNVs.

Initially, we tested three candidate gene lists for enrichment, based on the gene sets of the Gene Ontology Database (http://www.geneontology.org/). These gene sets represented three distinct neurobiological hypotheses on the etiology of RD (see the “Background” section): axon guidance (including all the GO sets containing the term “axon guidance”), neuronal migration (including all the GO sets containing the term “neuron migration”), and sex hormone biology (including all the GO sets containing the terms “steroid,” “androgen,” “estrogen,” “progesterone,” and “testosterone”). Then, we extended the assessment to 1748 GO sets containing at least 10 genes, for exploratory purposes.

## Results

### CNV calls

#### General CNV burden statistics

After QC, there were 4490 final CNV calls in 702 samples, of which 3344 were annotated to genes within a 50-kb interval from the UTRs, 2542 were annotated within exonic borders, and 872 were rare. Samples passing QC showed a median number of 6 CNVs per sample (4 considering only CNVs annotated to genes) and a median total length of ~640 kb covered by CNVs per sample (~479 kb considering only CNVs annotated to genes).

Correlation assessments between CNV burden measures (both CNV number and total length) and our continuous traits of interest—PC1 and IQadjPC1—did not reveal any significant correlation in the two CLDRC subsets, when considering all the CNVs passing QC (most significant correlation rho~−0.097, *p* = 0.4) or when considering only CNVs annotated to genes or to exons (most significant correlation rho~−0.036, *p* = 0.51). Similarly, burden analysis by length class (applied to all CNVs passing QC) did not show any significant correlation (most significant correlation rho~−0.17, *p* = 0.13, detected for short CNVs). We also tested correlation using burden statistics of rare CNVs called in our dataset, but again found no significant correlation with principal component (PC) scores (most significant correlation rho~−0.15, *p* = 0.2). Finally, case-control burden analysis comparing 67 RD cases and 69 controls did not reveal any significant association with CNV burden statistics (data not shown).

#### Large CNVs

Large CNVs are more likely to span multiple genes and to have deleterious effects than smaller CNVs. Among CNVs spanning more than 500 kb in RD cases (Table 2, see Additional file 2: Table S2a for further details), a heterozygous duplication was detected in two affected siblings, but not in their unaffected co-sibling (with IBGdiscr = −0.62, PC1 = −0.47, and IQadjPC1 = 0.42). This large CNV spanned ~1.2 Mb in the pericentromeric region 11q11-q12.1, covering several *OR* genes (encoding olfactory receptors) and *TRIM* genes (encoding tripartite motif proteins).

**Table 2:** Large annotated CNV events (>500 kb) detected in RD cases

Subject	Family	Chr	Start (kb)	End (kb)	SNPs	Length (kb)	CopyN	Frequency	Gene	PC1	IQadjPC1	IBGdiscr
IBG143157	3914	2	96,196	96,737	26	541	3	Common	FAHD2CP,GPAT2,LINC00342,TRIM43	−2.51	−2.59	−3.29
IBG112039	3576	11	48,397	48,943	33	546	1	Common	OR4A47	−0.59	−0.43	−1.76
IBG1448951	4442	14	19,848	20,420	17	573	3	Common	10 genes (including several OR genes)^a^	−0.97	−1.05	−2.83
IBG143577	4010	2	132,731	133,354	120	622	3	Common	ANKRD30BL,GPR39,MIR663B	−1.73	−1.54	−2.09
IBG112079	3906	8	105,737	106,407	147	670	3	Rare	ZFPM2	−1.56	−1.65	−1.98
IBG111829	2856	11	49,770	50,283	44	513	3	Common	LOC440040,LOC441601,OR4C12,OR4C13	−1.93	−1.74	−2.78
IBG112389	4048	5	45,672	46,399	35	727	3	Common	HCN1	−1.47	−1.57	−3.18
IBG145160	4499	11	54,794	56,004	190	1209	3	Common	30 genes (including several OR and TRIM genes)^b^	−2.21	−2.05	−3.63
IBG1451651	4499	11	54,794	56,004	190	1209	3	Common	30 genes (including several OR and TRIM genes)^b^	−1.83	−1.74	−2.14
IBG111948	3523	16	14,975	16,303	419	1328	3	Common	27 genes (including several microRNAs)^c^	−1.55	−1.53	−1.61

When a CNV is annotated to more than five RefSeq genes, these are reported in a footnote (see below). All the CNVs partially overlapped or encompassed the genes to which they were annotated. All the positions are expressed in hg19 coordinates. The frequency column specifies how each CNV call showed substantial overlaps (≥50 %) with any CNV reported in the DGV database (July 2013, hg19): ≥5 overlaps for common CNVs, <5 overlaps for rare CNVs. An extended format of this table including further details is available in Additional file 2: Table S2a)

^a^BMS1P17, BMS1P18, OR11H2, OR4K1, OR4K2, OR4K5, OR4M1, OR4N2, OR4Q3, POTEM

^b^OR10AG1, OR4A15, OR4A16, OR4C11, OR4C15, OR4C16, OR4C6, OR4P4, OR4S2, OR5AS1, OR5D13, OR5D14, OR5D16, OR5D18, OR5F1, OR5I1, OR5J2, OR5L1, OR5L2, OR5T2, OR5W2, OR7E5P, OR8H2, OR8H3, OR8I2, OR8J3, OR8K5, TRIM48, TRIM51, TRIM51HP

^c^ABCC1, ABCC6, C16orf45, FOPNL, KIAA0430, LOC100288162, MIR3179-1, MIR3179-2, MIR3179-3, MIR3180-1, MIR3180-2, MIR3180-3, MIR3180-4, MIR484, MIR6506, MIR6511A-2, MIR6511B-1, MIR6770-2, MPV17L, MYH11, NDE1, NOMO1, NPIPA1, NPIPA5, NTAN1, PDXDC1, RRN3

#### CNVs shared between RD cases

Among all the sibships analyzed, ten presented more than one RD case. In these sibships, we assessed annotated CNVs which were detected in two or more affected co-siblings but in no unaffected participant, regardless of their length. We investigated these variants as they were more likely to confer genetic susceptibility to reading impairment, compared to CNVs presented by single cases. A total of three CNV events fell in this category (Table 3, see Additional file 2: Table S2b for further details), including the large duplication mentioned above and other two CNV events.

**Table 3:** Annotated CNVs shared between two or more affected co-siblings in ten families presenting more than one RD case, which were not detected in any unaffected participant

Subject	Family	Chr	Start (kb)	End (kb)	SNPs	Length (kb)	CopyN	Frequency	Gene	PC1	IQadjPC1	IBGdiscr
IBG145160	4499	11	54,794	56,004	190	1209	3	Common	30 genes (including several OR and TRIM genes)^a^	−2.21	−2.05	−3.63
IBG1451651	4499	11	54,794	56,004	190	1209	3	Common	30 genes (including several OR and TRIM genes)^a^	−1.83	−1.74	−2.14
IBG142799	3514	6	145,148	145,175	15	27	3	Rare	UTRN	−1.62	−1.35	−2.12
IBG142797	3514	6	145,148	145,175	15	27	3	Rare	UTRN	−1.84	−2	−1.66
IBG142799	3514	1	225,391	225,454	14	63	3	Common	DNAH14	−1.62	−1.35	−2.12
IBG142797	3514	1	225,391	225,454	14	63	3	common	DNAH14	−1.84	−2	−1.66

When a CNV is annotated to more than five RefSeq genes, these are reported in a footnote (see below). All the CNVs partially overlapped or encompassed the genes to which they were annotated. All the positions are expressed in hg19 coordinates. The frequency column specifies how each CNV call showed substantial overlaps (≥50 %) with any CNV reported in the DGV database (July 2013, hg19): ≥5 overlaps for common CNVs, <5 overlaps for rare CNVs. An extended format of this table including further details is available in Additional file 2: Table S2b

^a^OR10AG1, OR4A15, OR4A16, OR4C11, OR4C15, OR4C16, OR4C6, OR4P4, OR4S2, OR5AS1, OR5D13, OR5D14, OR5D16, OR5D18, OR5F1, OR5I1, OR5J2, OR5L1, OR5L2, OR5T2, OR5W2, OR7E5P, OR8H2, OR8H3, OR8I2, OR8J3, OR8K5, TRIM48, TRIM51, TRIM51HP

In a family presenting two affected siblings but no unaffected co-siblings, we detected two shared CNVs (both heterozygous duplications), which were not detected in any other participant in the study. One of them, spanning ~27 kb on 6q24.2, covered the last nine exons (66–74) in the 3′ terminal region of the *UTRN* (utrophin) gene, including its 3′-UTR. The other one spanned ~63 kb and overlapped exons 38–49 within *DNAH14* (dynein axonemal heavy chain 14) on 1q42.12.

#### CNVs in genes previously associated with reading and language traits

We identified three putative CNVs annotated to candidate susceptibility genes that have been implicated in reading and language traits by more than one study (see [8–10] for reviews). These CNVs are reported in Additional file 2: Table S2c. Among the candidate genes assessed, *DYX1C1* and *CNTNAP2* were overlapped by one or more of these CNVs. However, only one of the three participants showing these variants was impaired and none of these CNVs co-segregated with poor reading-language performance (Additional file 2: Table S2c).

Similarly, we detected four CNV calls overlapping genes in which suggestive associations were observed in previous GWAS studies of both reading and language skills (reviewed in [10]). A list of these CNVs is reported in Additional file 2: Table S2d. Again, none of these variants co-segregated with RD status or with poor reading-language performance.

#### CNVs previously associated with weak reading/language performance and common neuropsychiatric CNVs

We checked our CNV calls for overlaps with genes and regions previously found to be disrupted by CNVs in subjects with weak reading/language performance (see the “Background” section). Additional file 2: Table S2e reports these CNVs, which were detected in *NEGR1*, *IMMP2L*, *PCDH11X*, *CNTNAP2*, *CSNK1A1*, *MSRA* (8p23.1), *UBASH3B*, *CACNA2D1*, *VWA3B*, *CXorf22*, *CHRNA7* (15q13.1), and in several genes in the 22q11.21 region. As before, none of these variants showed co-segregation with RD or poor reading-language performance in the sibships.

Similarly, we assessed overlaps with common neuropsychiatric CNVs recently tested by Stefansson and colleagues [31] for effects on several cognitive traits in a large sample of the Icelandic population. Additional file 2: Table S2f reports a list of canonical CNVs detected in our study (i.e., largely or completely overlapping the abovementioned neuropsychiatric CNVs, reported in Table S1 in [31]). Among these CNV events, a 1.33-Mb heterozygous duplication in 16p13.11 was detected in an affected participant, who had the lowest phenotypic scores in his sibship and exhibited strong score discrepancies with his co-sibling (>3 for IBGdiscr and PC1 and >2.6 for IQadjPC1). However, a similar duplication was present in an unrelated participant showing normal performance, with PC1 and IQadjPC1 scores higher than those of his sibling (data not shown). None of the other carriers of such canonical neuropsychiatric CNVs were RD cases, based on IBGdiscr performance (see Additional file 2: Table S2f).

When two or more CNV calls were overlapping in these regions, the encompassed probes were assessed in PLINK QFAM analysis of CNV state to detect stretches of consecutive probes associated with PC1 and IQadjPC1 scores.

### Family-based GWAS of principal component scores

#### Association test with CNV state at each probe

GWAS meta-analysis testing association between CNV state at each probe and PC1/IQadjPC1 did not report any significant association surviving correction for multiple testing of two traits and 5173 autosomal probes meta-analyzed (*α* = 4.8 × 10^−6^), representing all the probes encompassed by at least one putative CNV event in both our subsets. None of the 1900 X chromosome probes lay within CNV events detected in participants of both sexes and in both CLDRC subsets; therefore, none of these probes was meta-analyzed. The results of this analysis on an individual probe basis are reported in Additional file 3: Tables S3a, b. No genome-wide significant association was detected in either of the two subsets analyzed (data not shown).

These results were interpreted in terms of consecutive probes showing significant associations with PC1 and/or IQadjPC1 (i.e., at least two consecutive probes with *p* < 0.005 and contiguous with two or more probes with *p* < 0.05), in regions of overlap of two or more CNVs in our dataset (Table 4). All of the top associated regions showed nominally significant associations both with PC1 and IQadjPC1 (*p* values in the range [0.001; 0.05]), with the exception of chr3:2,663,757-2,675,189 (*p* values~[0.096; 0.245] in PC1 meta-analysis). All of these regions overlapped with genes: chr3:2,663,757-2,675,189 lay within *CNTN4* (contactin 4, 3p26.3; Additional file 3: Figure S3a); chr6:168,336,080-168,597,552 partially overlapped *MLLT4* (myeloid/lymphoid or mixed-lineage leukemia translocated to, 4) and encompassed the genes *KIF25* (kinesin family member 25), *FRMD1* (FERM domain containing 1), *KIF5-AS1* (KIF25 antisense RNA 1), and *GCH6.3* (uncharacterized protein) on 6q27 (Additional file 3: Figure S3b); chr10:68,221,549-68,242,672 lay within *CTNNA3* (catenin alpha 3, 10q21.3; Additional file 3: Figure S3c); and chr11:55,241,556-55,362,955 encompassed genes *OR4C15* and *OR4C16* (olfactory receptors 15 and 16, family 4, subfamily C, 11q11; Additional file 3: Figure S3d). Frequency of CNV+ state in these regions ranged between 0.4 and 3.4 %.

**Table 4:** Regions of CNV overlap showing the most significant associations with PC1/IQadjPC1 in the GWAS meta-analysis with CNV state (PLINK QFAM)

Chr	Start (bp)	Stop (bp)	Kb	SNPs	*p* value (PC1)	*p* value (IQadjPC1)	Effect^a^	Frequency (%)^b^	Gene^c^
3	2,663,757	2,675,189	11	13	[0.096; 0.245]	[0.003; 0.014]	+	0.4–0.6	CNTN4
6	168,336,080	168,597,552	261	130	[0.005; 0.176]	[0.001; 0.035]	−	1.7–3.4	MLLT4, KIF25, KIF25-AS1, HGC6.3, FRMD1
10	68,221,549	68,242,672	21	10	[0.015; 0.022]	[0.004; 0.007]	−	0.4	CTNNA3
11	55,241,556	55,362,955	121	28	[0.005; 0.013]	[0.002; 0.005]	−	0.4	OR4C15, OR4C16

All the regions of overlap of two or more CNVs, showing at least two consecutive probes with association *p* < 0.005 and two or more contiguous probes with association *p* < 0.05, are reported. The results of this meta-analysis on an individual probe basis are reported in detail in Additional file 3: Table S3a, b. All the positions are expressed in hg19 coordinates

^a^Effect of the CNV+ state, irrespective of the copy number, on PC1 and IQadjPC1

^b^Frequency (%) of the CNV+ state in the CLDRC dataset

^c^Refseq genes overlapped/encompassed by the region reported. None of these regions annotated to potentially regulatory elements in the genome, such as transcription factor binding sites, digital DNase I hypersensitivity clusters, and H3K27Ac histone marks, as collected in ENCODE tracks. Similarly, no annotation was detected to the most conserved genomic regions in the primate clade (PhastCons 46-way elements), nor to the most positively selected regions since Homo Sapiens-Neanderthal split (i.e., regions showing S scores from Selective Sweep Scan track in the lower 5 %). All the tracks used here are available for download from the UCSC table browser (http://genome.ucsc.edu/cgi-bin/hgTables)

**Fig. 2:**
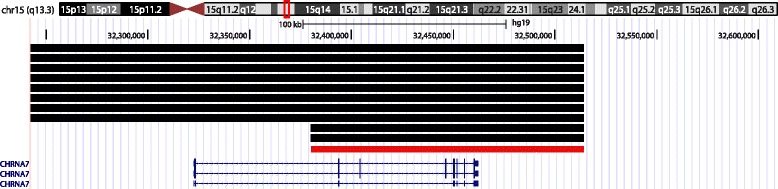
Candidate region of CNV overlap associated with PC1 in the GWAS with CNV state. The *red line* indicates the associated interval chr15:32,380,064-32,514,341, partially overlapping *CHRNA7* (15q13.3). *Black horizontal lines* represent the individual CNV calls detected in this region (nine heterozygous duplications and one heterozygous deletion; see Additional file 2: Table S2e for details)

We also checked the presence of nominally significant associations (i.e., at least two consecutive probes with *p* < 0.05) in the regions disrupted by CNVs in cases of reading, language, or more severe neuropsychiatric disorders (see the “Background” section). If CNV events in any of these regions had been called only in one of the subsets and therefore meta-analysis had not been run for the probes encompassed, we assessed directly the GWAS results in the relevant subset. Among the candidate CNVs assessed, a ~134-kb region (chr15:32,380,064-32,514,341) partially overlapping *CHRNA7* (cholinergic nicotinic receptor alpha 7, 15q13.3; Fig. 2) showed a series of 25 consecutive probes associated with PC1 (*p* values~[0.026; 0.045], Additional file 3: Table S3e). These associations were detected in CLDRC-RD as no CNVs were called in the CLDRC-ADHD subset and were not significant in the IQadjPC1 GWAS (*p* values~[0.053; 0.085]). This region showed a frequency of CNV+ state of ~1.8 % (see Additional file 2: Table S2e for relevant CNV calls) and a positive allelic trend between the CNV+ state and PC1/IQadjPC1 (see Additional file 3: Table S3e).

#### Association test with probe intensity data

GWAS meta-analysis of PC1/IQadjPC1 scores with intensity data (FamCNV) did not reveal any genome-wide significant association surviving correction for multiple testing of 704,855 autosomal probes and two traits meta-analyzed (*α* = 3.6 × 10^−8^). The results of this analysis on an individual probe basis are reported in Additional file 3: Tables S3c, d. No genome-wide significant association was detected in the two subsets analyzed (data not shown).

**Fig. 3:**
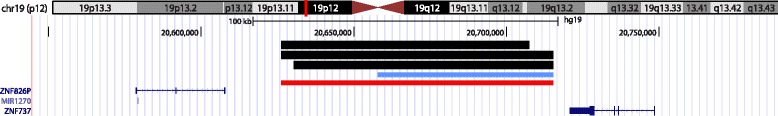
19p12 region associated with PC traits in the GWAS with probe intensity data. The *blue line* indicates the interval of association with probe intensity data (chr19:20,657,781-20,715,228), while the *red line* indicates the overlapping region of association between CNV state and PC scores (chr19:20,626,179-20,715,228). *Black horizontal lines* represent the three types of deletions detected in this region (reported in Additional file 2: Table S2g)

Also for this analysis, we were interested in detecting two or more consecutive probes showing significant association. For this purpose, we filtered our association results to detect all the pairs of consecutive probes with *p* < 0.001 and contiguous with two or more probes with *p* < 0.05. Such criteria were set to reduce the probability to observe spurious associations due to noise intrinsic to raw intensity data. Although we did not find any region meeting these criteria in the results of the meta-analysis, we found such a region in the GWAS in CLDRC-RD. This ~58-kb region (chr19:20,657,781-20,715,228) consisted of eight consecutive SNPs on 19p12, associated with both PC1 (top consecutive hits rs2021399 and rs2545918, *p* = 6 × 10−^4^ and 4 × 10^−4^, respectively) and IQadjPC1 (*p* = 2 × 10^−4^ and 9 × 10^−4^; see Additional file 3: Table S3f). This region lay within a ~80-kb deletion that was frequent in our dataset (called in 11.3 % of CLDRC participants, for a total of 80 CNV calls, reported in Additional file 2: Table S2g) and ~6-kb downstream of *ZNF737* (zinc finger protein 737, Fig. 3). The same region of overlap also showed nominally significant association (*p* values~[0.009; 0.022]) in the PLINK QFAM analysis with CNV state, in a wider interval (chr19:20,626,179-20,715,228, see Fig. 3 and Additional file 3: Table S3g). Both in the association test with SNP intensity data and in the association test with CNV state, this deletion showed a positive effect on PC1/IQadjPC1.

### Pathway-based analysis of CNV calls

Pathway association analysis of 306 CNV calls presented by 67 RD cases did not reveal any significant enrichment, neither in the analysis of three composite candidate pathways representing neuronal migration, axonal guidance, and steroids-related processes (Additional file 3: Table S3h), nor in an exploratory analysis at the pathway-wide level (data not shown). Similarly, we did not observe any significant enrichment in the analysis of 84 rare CNVs detected in RD cases (see Additional file 3: Table S3i for results on candidate pathways).

## Discussion

Our research on potential effects of CNVs on reading and language is novel for two main reasons:

First, we investigated the effects of CNVs on a continuous index of reading and language performance, in datasets enriched for the lower tail of the population distribution. Although a similar approach was used in a recent study by Stefansson and colleagues [31], who investigated the effect of candidate neuropsychiatric CNVs on cognitive traits in a large sample of the Icelandic population, their study analyzed a broad spectrum of cognitive abilities and included general population controls. It was not aimed at capturing shared variation derived from a detailed battery of reading and language measures in a selected population, as was our study.

Second, to detect effects of CNVs on continuous reading and language performance, we used two complementary approaches: one aimed at detecting copy number-dependent effects in a “dosage-dependent” additive model and one aimed at detecting associations with a “CNV-positive” state irrespective of the non-diploid copy number. These two analyses were performed in order to identify potential CNVs with reduced penetrance and variable expressivity and were in turn complementary to our analysis of co-segregation between CNVs and RD status, which was aimed at detecting variants with high penetrance and expressivity.

In our dataset of subjects with school histories of RD/ADHD and their siblings, we did not identify a significant correlation between CNV genomic burden—both in terms of total length and of total number of CNVs per subject—and PC scores representing reading-language performance. Similarly, our case-control burden analysis did not reveal any significant contribution of CNVs to RD. This is in line with a previous study which detected no significant difference in the genomic burden of large rare CNVs between RD cases and controls [20]. However, our result is in partial contrast with a recent study which reported an increased CNV burden in SLI cases compared to controls [23]. On balance, it appears likely that CNVs have a relatively limited role in affecting reading-related performance at the population level, whereas they are known to play a more important role in severe neuropsychiatric disorders such as autism, schizophrenia, and ID [15, 16, 20] and may also affect severe cases of SLI. Further analyses in independent datasets will be needed to clarify the extent to which CNVs may affect cognitive domains that are shared between reading and language.

In this study, we detected a CNV which co-segregated with the dyslexic status in a family with two RD cases—including the most severely impaired subject in our dataset—and one unaffected sibling. This large CNV event spanned ~1.2 Mb in the pericentromeric region 11q11-q12.1, covering several *OR* (olfactory receptors) and *TRIM* (tripartite motif protein) genes. While TRIM proteins are not well characterized, the role of olfactory receptors in triggering odor perception signals in sensory neurons is well known. Interestingly, olfactory bulbs dysgenesis/agenesis has been previously implicated in ASD [43] and reduced volumes have been reported in schizophrenic patients [44]. However, the partial overlap of this CNV with a centromeric region and the relaxed selection at the *OR* loci [45] suggest caution in the biological interpretation of this variant.

Two other CNVs shared between cases were detected, overlapping potential susceptibility genes. These two heterozygous duplications were observed in a pair of affected siblings but were not detected in any unaffected participant. One of them overlapped 9 exons in the 3′ terminal region of the *UTRN* gene (utrophin, or dystrophin-related protein 1, 6q24.2) and the other one overlapped 12 exons within *DNAH14* (dynein axonemal heavy chain 14, 1q42.12). Utrophin is a large skeletal muscle protein—also expressed in the CNS—contributing to postsynaptic membrane maintenance and to clustering of acetylcholine receptors in the neuromuscular synapses and possibly playing a role in anchoring the cytoskeleton to the plasma membrane. However, as the partial duplication of *UTRN* overlaps its 3′-UTR region, it is possible that this has no effect on the mRNA produced. This possibility may be addressed through future gene functional analysis. Dyneins are microtubule-associated motor proteins with a key role in cilia-mediated cell motility. Independent studies have reported evidence of involvement in cilia-related processes for two RD candidate genes, *DYX1C1* [46, 47] and *DCDC2* [48]. This led to the hypothesis that dyslexia may sometimes be a form of ciliopathy [46], involving abnormal neuronal development and migration [48].

Pathway-based enrichment testing of CNV calls detected in RD cases revealed no significant associations for three candidate gene sets representing mainstream hypotheses on the etiology of RD, namely axon guidance, neuron migration, and steroids-related processes. This is in line with the pathway enrichment test based on SNP associations in an earlier GWASMA study that we carried out [11].

The two complementary strategies for genome-wide association testing between CNVs and our principal component reading-language scores revealed partly different but partly consistent results. The first of these analyses—which made use of CNV calls and tested association with CNV state at each probe—was aimed at detecting associations in regions of overlap of CNV calls, irrespective of the abnormal copy number state. The second analysis, in FamCNV, assessed copy number (or allele dosage)-dependent associations between DNA array intensity data and PC1/IQadjPC1. These are practical strategies to detect different kinds of effects of CNVs on continuous traits, both of which have precedence in the literature: in recent studies, copy number-dependent (dosage) effects were reported for continuous traits such as body mass index [49] and structural brain measures [31]; while either deletions or reciprocal duplications of specific regions have been reported to result in similar clinical and phenotypic manifestations, as in the case of ASD, language/developmental delays, and other psychiatric disorders [15, 16]. Both analyses were run probe-by-probe, but results were then interpreted in terms of consecutive probes showing significant associations, which was appropriate for an analysis of CNVs.

Although no associations reached genome-wide significance in PLINK QFAM meta-analysis, some of the top associated regions involve plausible candidate genes. A ~11-kb CNV overlap, associated with IQadjPC1, lay in an intronic region within *CNTN4* (contactin 4, 3p26.3; Additional file 3: Figure S3a). This overlap was shared by three heterozygous duplications and one heterozygous deletion, which all showed concordant positive effects on PC scores. Contactins are Ig cell adhesion molecules with a fundamental role in neuronal development and plasticity. CNVs and structural rearrangements disrupting *CNTN4* have been implicated in severe neurodevelopmental disorders such as ASD [50, 51] and DD [52]. Interestingly, the associated region detected in the present study overlaps with CNVs reported in ASD cases in two previous studies [50, 51], and contactin 4 is widely expressed in the brain, particularly in the cerebellum, thalamus, amygdala, and cerebral cortex [50]. However, this association was weaker and not significant with PC1.

Another intronic CNV overlap region of ~21 kb, associated with both PC1 and IQadjPC1, was found within *CTNNA3* (catenin alpha 3, 10q21.3; Additional file 3: Figure S3c). This region resulted from the overlap of three deletions and showed a negative effect on PC scores. α-catenins have a crucial role in cell adhesion, and *CTNNA3* has been implicated in ASD etiology through both CNV studies [53, 54] and GWAS studies [55, 56]. Our associated region partially overlaps an inherited compound heterozygous deletion encompassing exon 11, found in an ASD patient [53]. Expression of *CTNNA3* in mouse hippocampus and cortex at postnatal day 0 suggests a specific neuronal role at very early developmental stages [53].

We also assessed CNV overlaps in regions previously reported to be disrupted by CNVs in reading, language, or more severe neuropsychiatric disorders. Among these, a ~134-kb region of overlap between nine heterozygous duplications and one heterozygous deletion, encompassing several exons in the 3′ region of *CHRNA7* (cholinergic nicotinic receptor alpha 7, 15q13.3; Fig. 2), presented nominally significant association with PC1 in the CLDRC-RD subset (while no CNV calls were detected in CLDRC-ADHD). The association only approached significance after IQ adjustment and the CNV state exerted a positive effect on PC1/IQadjPC1, with both deletion and duplications showing the same direction of effect. Nicotinic cholinergic receptors are ligand-gated ion channels that mediate fast signal transmission at synapses and are ubiquitously expressed in the CNS. Several studies have suggested a possible involvement of *CHRNA7* in language skills. A CNV encompassing this gene was suggested to contribute to the disruption of synaptic pathways in a patient with ID and language impairment [57]. A genome-wide CNV screen also reported *CHRNA7* among the genes disrupted in a group of unrelated SLI cases, as well as a significant over-representation of the GO category *acetylcholine binding* in a pathway-based analysis of these CNVs [23]. A recent longitudinal study of children with language difficulties implicated a deletion at 15q13.1-13.3 (BP3-BP5) in the etiology of SLI, and the authors hypothesized a role of *CHRNA7* in the phenotypic effects associated to this region [24]. The 15q13.3 region is also a hotspot of neuropsychiatric CNVs, which have been implicated in several disorders including SCZ, ASD, ADHD, and epilepsy [15, 16]. CNVs encompassing this gene have also been tested for effects on general cognitive abilities, including school history of mathematical and reading difficulties, but no associations were reported [31].

Similarly to PLINK QFAM analysis, FamCNV meta-analysis did not reveal any genome-wide significant association. However, we found a series of eight contiguous SNPs associated with both PC1 and IQadjPC1 in the CLDRC-RD analysis, ~6-kb downstream of *ZNF737* (zinc finger protein 737, 19p12, Fig. 3). This ~58-kb region lay within a ~80-kb deletion which was common in our dataset, and the association was also observed at the nominal significance level in the PLINK QFAM analysis of CLDRC-RD. Both FamCNV and QFAM analysis indicated a positive effect of this deletion on PC1/IQadjPC1. Zinc finger protein 737 has not been functionally characterized, but the presence of a zinc finger domain suggests a possible involvement in transcriptional regulation. Interestingly, a microdeletion within another zinc finger gene, *ZNF277*, has been suggested as susceptibility CNV for SLI [22].

In spite of the interesting suggestive associations discussed above, the modest sample size and absence of a replication sample constitute limitations for the present study, and further analyses in larger datasets are warranted. In addition, the localization of CNV breakpoints and functional validation of candidate CNVs can help to validate and extend such associations. Also, the definition of RD cases was necessarily somewhat arbitrary (as in all studies). Nonetheless, for completeness of our analysis, we used this approach to assess co-segregation with CNVs in the sibships. As there is no universal agreement on the diagnostic definition of dyslexia [1, 3], we used a “performance only”-based criterion, classifying all the participants in the lowest 10 % of the IBGdiscr score distribution as RD cases and considering them as representative of reading impairment. Finally, it may be observed that many of the CNVs identified are in CNV hotspot regions and they may represent benign variants. Nonetheless, the fact that these CNVs are frequently detected in the general population does not rule out potentially modifying effects on reading and language skills. For this reason, we did not exclude CNVs present in the DGV from our analysis.

## Conclusions

Overall, this study did not identify clear effects of CNVs on reading and language performance, but identified a number of putative, individual susceptibility factors in the genome. We believe that applying the comprehensive strategy used in this study to larger datasets may facilitate the identification of new structural variants involved in reading and language performance and reveal part of the missing heritability for reading and language measures.

## Additional files

Additional file 1: Supplementary methods. Details on IBG discriminant score; pairwise phenotypic correlations of PC1, IQadjPC1, and IBGdiscr; principal component analysis of LRR intensity data from DNA array. (DOCX 50 kb) Additional file 2: CNV calls of interest. Large CNV calls; CNVs shared between two or more affected co-siblings (extended tables); CNV calls within genes implicated in reading and language; CNV calls overlapping genes implicated in reading and/or language impairments by previous CNV studies; CNV calls overlapping common neuropsychiatric CNVs; CNV calls annotated to *ZNF737*. (XLSX 41 kb) Additional file 3: Supplementary results. Detailed results of FamCNV and PLINK QFAM GWAS meta-analyses; details of associations in the *CHRNA7* and *ZNF737* regions; results of pathway-based analysis. (DOCX 89 kb)
