# Supplementary material for: Investigating the effects of copy number variants on reading and language performance
Source: J Neurodev Disord. 2016 May 15;8:17. doi: 10.1186/s11689-016-9147-8 (PMC4868026; doi:10.1186/s11689-016-9147-8)
Supplement: Additional file 1: — Supplementary methods. Details on IBG discriminant score; pairwise phenotypic correlations of PC1, IQadjPC1, and IBGdiscr; principal component analysis of LRR intensity data from DNA array. (DOCX 50 kb) [file 11689_2016_9147_MOESM1_ESM.docx]

***Supplementary Methods***

**IBG discriminant score**

The IBG discriminant score (called *IBGdiscr* hereafter) is a discriminant function empirically developed by John DeFries [[1](#_ENREF_1)] at the Institute of Behavioral Genetics of University of Colorado at Boulder. This was obtained from the analysis of an independent sample of 140 reading-disabled and 140 control children [[1](#_ENREF_1)], to diagnose dyslexia in the context of the CLDRC study. This function is a composite measure of word recognition, spelling and comprehension subtests of the Peabody Individual Achievement Test [[2](#_ENREF_2)], as detailed in the formula below:

IBGdiscr = 1.48121 + 0.078432 * WRead + 0.4810 * WSpell + 0.03453 * WComp,

where WRead and WSpell are measures of word reading and spelling (further details in [[3](#_ENREF_3)]) and WComp is a measure of reading comprehension obtained through a multiple choice test, statistically elaborated in the same way as the other two measures (i.e. adjusted for age and age^2^ and standardized against the normative mean of a control population).

a)

b)

c)

**Figure S1.** IBG discriminant score distributions in the **a)** CLDRC dataset (N=702 after CNV calling process), **b)** CLDRC-RD (N=543) and **c)** CLDRC-ADHD subset (N=159).

| **Trait** | PC1 | IQadjPC1 | IBGdiscr |
| --- | --- | --- | --- |
| PC1 | 1 | 0.94 | 0.92 |
| IQadjPC1 | 0.98 | 1 | 0.84 |
| IBGdiscr | 0.87 | 0.83 | 1 |

**Table S1.** Pairwise phenotypic correlations of PC1, IQadjPC1 and IBGdiscr in CLDRC-RD (above the diagonal) and CLDRC-ADHD (below the diagonal). These were computed as median Pearson's r coefficients over 100 repeat random samplings of one individual from each unrelated sibship, separately within each subset.


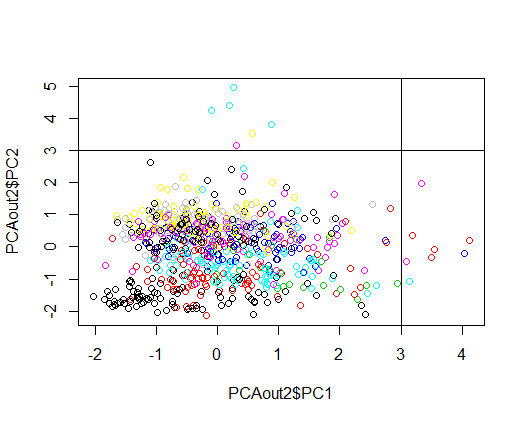


**Figure S1d.** Scatter plot showing the first two components extracted from the PCA analysis of LRR intensity data of 723,002 SNPs, run on 727 subjects passing genotype and phenotype QC in our previous GWAS meta-analysis [[3](#_ENREF_3)]. The first (PC1) and second principal component (PC2) explained 30% and 10% of the total variance in LRR data, while the remaining 98 component scores represented no more than 3% of the total variance each. Samples are colored differently based on the DNA array plate of belonging, in order to detect any potential batch effect among different experiments. Outliers were defined as samples showing scaled PC score >3 for any of the first two principal components, or a PC score >2 for both PC1 and PC2.

### References

### 1. DeFries JC: Colorado Reading Project. In: Parkton, MD: York Press; 1985: 107-122

### 2. Dunn LM, Markwardt FC: *Examiner’s Manual: Peabody Individual Achievement Test.*: American Guidance Service: Circle Pines, MN.; 1970.

### 3. Gialluisi A, Newbury DF, Wilcutt EG, Olson RK, DeFries JC, Brandler WM, Pennington BF, Smith SD, Scerri TS, Simpson NH, et al: Genome-wide screening for DNA variants associated with reading and language traits. *Genes, Brain and Behavior* 2014, 13:686-701.
