# Supplementary material for: Investigating the effects of copy number variants on reading and language performance
Source: J Neurodev Disord. 2016 May 15;8:17. doi: 10.1186/s11689-016-9147-8 (PMC4868026; doi:10.1186/s11689-016-9147-8)
Supplement: Additional file 3: — Supplementary results. Detailed results of FamCNV and PLINK QFAM GWAS meta-analyses; details of associations in the CHRNA7 and ZNF737 regions; results of pathway-based analysis. (DOCX 89 kb) [file 11689_2016_9147_MOESM3_ESM.docx]

***Additional file 3: Supplementary Results***

S3a)

| Chr | SNP | Position (hg19) | Zscore | P-value | Direction^a^ | HetPVal^b^ | Gene (distance)^c^ |
| --- | --- | --- | --- | --- | --- | --- | --- |
| 5 | rs283107 | 32101400 | 2.913 | 3.6 x 10-3 | ++ | 0.59 | PDZD2(0)\|GOLPH3(+23.42) |
| 6 | rs12198918 | 168593739 | -2.815 | 4.9 x 10-3 | -- | 0.83 |  |

S3b)

| Chr | SNP | Position (hg19) | Zscore | P-value | Direction^a^ | HetPVal^b^ | Gene (distance)^c^ |
| --- | --- | --- | --- | --- | --- | --- | --- |
| 3 | rs2170004 | 2665743 | 2.825 | 4.7 x 10-3 | ++ | 0.3 | CNTN4(0) |
| 3 | rs1904396 | 2667880 | 2.919 | 3.5 x 10-3 | ++ | 0.34 | CNTN4(0) |
| 3 | rs2600317 | 2669957 | 2.807 | 5 x 10-3 | ++ | 0.33 | CNTN4(0) |
| 3 | rs17017512 | 2670066 | 2.835 | 4.6 x 10-3 | ++ | 0.34 | CNTN4(0) |
| 3 | rs2728071 | 2672217 | 2.85 | 4.4 x 10-3 | ++ | 0.3 | CNTN4(0) |
| 3 | rs6770875 | 2672936 | 2.862 | 4.2 x 10-3 | ++ | 0.35 | CNTN4(0) |
| 5 | rs283107 | 32101400 | 3.066 | 2.2 x 10-3 | ++ | 0.77 | PDZD2(0)\|GOLPH3(+23.42) |
| 6 | rs6455492 | 168578718 | -2.832 | 4.6 x 10-3 | -- | 0.58 |  |
| 6 | rs7751205 | 168579302 | -3.177 | 1.5 x 10-3 | -- | 0.81 |  |
| 6 | rs4708445 | 168580694 | -3.296 | 9.8 x 10-4 | -- | 0.78 |  |
| 6 | rs11960954 | 168580741 | -3.377 | 7.3 x 10-4 | -- | 0.78 |  |
| 6 | rs9455968 | 168581362 | -3.164 | 1.6 x 10-3 | -- | 0.7 |  |
| 6 | rs9455971 | 168582182 | -3.268 | 1.1 x 10-3 | -- | 0.79 |  |
| 6 | rs9455973 | 168583006 | -3.278 | 1 x 10-3 | -- | 0.79 |  |
| 6 | rs2880102 | 168583032 | -3.197 | 1.4 x 10-3 | -- | 0.78 |  |
| 6 | rs9355178 | 168589242 | -3.169 | 1.5 x 10-3 | -- | 0.65 |  |
| 6 | rs9283861 | 168592134 | -3.201 | 1.4 x 10-3 | -- | 0.71 |  |
| 6 | rs12198918 | 168593739 | -3.309 | 9.3 x 10-4 | -- | 0.73 |  |
| 6 | rs9346533 | 168593956 | -3.229 | 1.2 x 10-3 | -- | 0.75 |  |
| 6 | rs12213783 | 168595832 | -3.254 | 1.1 x 10-3 | -- | 0.68 |  |
| 10 | rs2921945 | 68221549 | -2.836 | 4.6 x 10-3 | -- | 0.91 | CTNNA3(0) |
| 10 | rs4745900 | 68224593 | -2.863 | 4.2 x 10-3 | -- | 0.89 | CTNNA3(0) |
| 10 | rs2441727 | 68224886 | -2.842 | 4.5 x 10-3 | -- | 0.9 | CTNNA3(0) |
| 10 | rs12220315 | 68225548 | -2.822 | 4.8 x 10-3 | -- | 0.9 | CTNNA3(0) |
| 10 | rs11817581 | 68237143 | -2.85 | 4.4 x 10-3 | -- | 0.91 | CTNNA3(0) |
| 10 | rs10822837 | 68242672 | -2.876 | 4 x 10-3 | -- | 0.85 | CTNNA3(0) |
| 11 | rs4537777 | 55241556 | -3.044 | 2.3 x 10-3 | -- | 0.74 |  |
| 11 | rs534345 | 55256498 | -2.989 | 2.8 x 10-3 | -- | 0.79 |  |
| 11 | rs17158615 | 55258370 | -2.985 | 2.8 x 10-3 | -- | 0.75 |  |
| 11 | rs10896971 | 55264310 | -2.893 | 3.8 x 10-3 | -- | 0.74 |  |
| 11 | rs12272148 | 55272791 | -3.003 | 2.7 x 10-3 | -- | 0.69 | OR4C15(-48.99) |
| 11 | rs559362 | 55275456 | -2.998 | 2.7 x 10-3 | -- | 0.77 | OR4C15(-46.33) |
| 11 | rs12417844 | 55282064 | -2.993 | 2.8 x 10-3 | -- | 0.77 | OR4C15(-39.72) |
| 11 | rs17159005 | 55303865 | -2.858 | 4.3 x 10-3 | -- | 0.76 | OR4C16(-35.74)\|OR4C15(-17.92) |
| 11 | rs526821 | 55306151 | -3.058 | 2.2 x 10-3 | -- | 0.73 | OR4C16(-33.45)\|OR4C15(-15.63) |
| 11 | rs17580938 | 55311980 | -2.99 | 2.8 x 10-3 | -- | 0.73 | OR4C16(-27.62)\|OR4C15(-9.802) |
| 11 | rs504661 | 55312683 | -2.978 | 2.9 x 10-3 | -- | 0.77 | OR4C16(-26.92)\|OR4C15(-9.099) |
| 11 | rs17581191 | 55316023 | -2.9 | 3.7 x 10-3 | -- | 0.73 | OR4C16(-23.58)\|OR4C15(-5.759) |
| 11 | rs509882 | 55321055 | -2.976 | 2.9 x 10-3 | -- | 0.77 | OR4C16(-18.55)\|OR4C15(-0.727)\|OR4C11(+49.86) |
| 11 | rs12790125 | 55322539 | -3.003 | 2.7 x 10-3 | -- | 0.74 | OR4C16(-17.06)\|OR4C15(0)\|OR4C11(+48.38) |
| 11 | rs17581700 | 55322606 | -3 | 2.7 x 10-3 | -- | 0.75 | OR4C16(-17)\|OR4C15(0)\|OR4C11(+48.31) |
| 11 | rs12225462 | 55322638 | -2.935 | 3.3 x 10-3 | -- | 0.81 | OR4C16(-16.96)\|OR4C15(0)\|OR4C11(+48.28) |
| 11 | rs506988 | 55325928 | -3.035 | 2.4 x 10-3 | -- | 0.7 | OR4C16(-13.68)\|OR4C15(+3.033)\|OR4C11(+44.99) |
| 11 | rs1394428 | 55335878 | -2.959 | 3.1 x 10-3 | -- | 0.79 | OR4C16(-3.725)\|OR4C15(+12.98)\|OR4C11(+35.04) |
| 11 | rs1459101 | 55339652 | -2.992 | 2.8 x 10-3 | -- | 0.69 | OR4C16(0)\|OR4C15(+16.76)\|OR4C11(+31.26) |
| 11 | rs558465 | 55339748 | -2.908 | 3.6 x 10-3 | -- | 0.82 | OR4C16(0)\|OR4C15(+16.85)\|OR4C11(+31.17) |
| 11 | rs557590 | 55339829 | -2.898 | 3.8 x 10-3 | -- | 0.77 | OR4C16(0)\|OR4C15(+16.93)\|OR4C11(+31.09) |
| 11 | rs559449 | 55340379 | -2.816 | 4.9 x 10-3 | -- | 0.86 | OR4C16(0)\|OR4C15(+17.48)\|OR4C11(+30.54) |
| 11 | rs35992551 | 55340631 | -3.039 | 2.4 x 10-3 | -- | 0.72 | OR4C16(+0.095)\|OR4C15(+17.74)\|OR4C11(+30.29) |
| 11 | rs12421826 | 55343036 | -2.955 | 3.1 x 10-3 | -- | 0.78 | OR4C16(+2.5)\|OR4C15(+20.14)\|OR4C11(+27.88) |
| 11 | rs2903854 | 55360213 | -2.848 | 4.4 x 10-3 | -- | 0.8 | OR4P4(-45.62)\|OR4C16(+19.68)\|OR4C15(+37.32)\|OR4C11(+10.7) |
| 11 | rs546140 | 55361808 | -2.959 | 3.1 x 10-3 | -- | 0.76 | OR4P4(-44.02)\|OR4C16(+21.27)\|OR4C15(+38.91)\|OR4C11(+9.108) |
| 11 | rs578686 | 55362955 | -3.009 | 2.6 x 10-3 | -- | 0.73 | OR4P4(-42.88)\|OR4C16(+22.42)\|OR4C15(+40.06)\|OR4C11(+7.961) |

**Table S3.** Top associated probes (p < 0.005) in the GWAS meta-analysis of **a)** PC1 and **b)** IQ-adjusted PC1with CNV state (implemented in PLINK QFAM). Genome-wide significance threshold: α = 4.8x10^-6^, corrected for multiple testing of two PC scores and ~5,173 SNPs encompassed by at least one putative CNV event in both CLDRC-RD and CLDRC-ADHD. Probes are ordered by chromosome and position to facilitate the interpretation of results in terms of consecutive probes associated.

^a^ The direction of effect refers to the "CNV+" state (i.e. copy number other than 2) and is reported for subsets in the following order: CLDRC-RD, CLDRC-ADHD. ^b^ Test for the homogeneity of effect sizes across the different subsets (p ≥ 0.05 indicates homogeneous effects). ^c^ Physical distance (kb) from close genes (in a ±50kb range from each marker) is indicated, along with orientation based on the direction of transcription ("-" = upstream of 5'-UTR, "+" = downstream of 3'-UTR).

S3c)

| Chr | SNP | Position (hg19) | Zscore | P-value | Direction^a^ | HetPVal^b^ | Gene (distance)^c^ |
| --- | --- | --- | --- | --- | --- | --- | --- |
| 12 | rs7295708 | 13455190 | -4.816 | 1.5 x 10-6 | -+ | 0.003524 |  |
| 1 | rs6429178 | 240225460 | 4.78 | 1.8 x 10-6 | ++ | 0.9811 | FMN2(-29.72) |
| 7 | rs7779972 | 138746752 | 4.662 | 3.1 x 10-6 | ++ | 0.8939 | ZC3HAV1(0)\|ZC3HAV1L(-25.98) |
| 16 | rs12149867 | 46984457 | 4.544 | 5.5 x 10-6 | ++ | 0.1318 | GPT2(+19.26)\|DNAJA2(+4.816) |
| 3 | rs6776460 | 107597927 | -4.533 | 5.8 x 10-6 | -- | 0.06897 |  |
| 4 | rs10018177 | 167020324 | 4.527 | 6 x 10-6 | ++ | 0.5812 | TLL1(0) |
| 17 | rs6502435 | 15072464 | 4.522 | 6.1 x 10-6 | ++ | 0.856 |  |
| 2 | rs7425117 | 100445030 | 4.517 | 6.3 x 10-6 | ++ | 0.5408 | AFF3(0) |
| 13 | rs7318170 | 106659814 | -4.478 | 7.5 x 10-6 | -- | 0.7184 |  |
| 1 | rs1016090 | 34924784 | 4.438 | 9.1 x 10-6 | ++ | 0.08746 |  |
| 12 | rs7300680 | 130299753 | -4.428 | 9.5 x 10-6 | -- | 0.6741 | TMEM132D(0) |

S3d)

| Chr | SNP | Position (hg19) | Zscore | P-value | Direction^a^ | HetPVal^b^ | Gene (distance)^c^ |
| --- | --- | --- | --- | --- | --- | --- | --- |
| 16 | rs12149867 | 46984457 | 4.567 | 4.9 x 10-6 | ++ | 0.07557 | GPT2(+19.26)\|DNAJA2(+4.816) |
| 1 | rs7518950 | 21804457 | 4.518 | 6.3 x 10-6 | ++ | 0.5933 | NBPF3(0)\|ALPL(-31.4) |
| 2 | rs7572476 | 242496325 | -4.516 | 6.3 x 10-6 | -- | 0.1711 | THAP4(+27.49)\|STK25(-48.29)\|BOK(-1.866) |
| 3 | rs11921628 | 149787516 | 4.475 | 7.6 x 10-6 | ++ | 0.247 |  |
| 12 | rs7295708 | 13455190 | -4.426 | 9.6 x 10-6 | -+ | 0.01375 |  |

**Table S3.**Top associated probes (p < 1x10^-5^) in the GWAS meta-analysis of **c)** PC1 and **d)** IQ-adjusted PC1 with probe intensity data (implemented in FamCNV). Genome-wide significance threshold: α = 3.6x10^-8^, corrected for multiple testing of two PC scores and 704,855 autosomal probes.

^a^ The direction of effect refers to the rho correlation coefficient between the LRR intensity signal and the relevant PC score and is reported for subsets in the following order: CLDRC-RD, CLDRC-ADHD. ^b^ Test for the homogeneity of effect sizes across the different subsets (p ≥ 0.05 indicates homogeneous effects). ^c^ Physical distance (kb) from close genes (in a ±50kb range from each marker) is indicated, along with orientation based on the direction of transcription ("-" = upstream of 5'-UTR, "+" = downstream of 3'-UTR).

S3e)

| Chr | SNP | Position (hg19) | Beta (PC1)^a^ | P-value (PC1) | Beta (IQadjPC1)^a^ | P-value (IQadjPC1) | Gene (distance)^b^ |
| --- | --- | --- | --- | --- | --- | --- | --- |
| 15 | rs1399190 | 32380064 | 0.76 | 0.042 | 0.64 | 0.082 | CHRNA7(0) |
| 15 | rs8033518 | 32381609 | 0.76 | 0.031 | 0.64 | 0.064 | CHRNA7(0) |
| 15 | rs6494212 | 32385119 | 0.76 | 0.026 | 0.64 | 0.077 | CHRNA7(0) |
| 15 | rs7175581 | 32385467 | 0.76 | 0.036 | 0.64 | 0.053 | CHRNA7(0) |
| 15 | rs7179082 | 32386214 | 0.76 | 0.037 | 0.64 | 0.077 | CHRNA7(0) |
| 15 | rs8036104 | 32389362 | 0.76 | 0.037 | 0.64 | 0.063 | CHRNA7(0) |
| 15 | rs4779565 | 32390070 | 0.76 | 0.033 | 0.64 | 0.065 | CHRNA7(0) |
| 15 | rs8035668 | 32391346 | 0.76 | 0.032 | 0.64 | 0.064 | CHRNA7(0) |
| 15 | rs12440480 | 32391391 | 0.76 | 0.033 | 0.64 | 0.066 | CHRNA7(0) |
| 15 | rs6494223 | 32396457 | 0.76 | 0.043 | 0.64 | 0.073 | CHRNA7(0) |
| 15 | rs8028396 | 32396721 | 0.76 | 0.027 | 0.64 | 0.06 | CHRNA7(0) |
| 15 | rs10438342 | 32402046 | 0.76 | 0.028 | 0.64 | 0.062 | CHRNA7(0) |
| 15 | rs11858834 | 32402921 | 0.76 | 0.03 | 0.64 | 0.073 | CHRNA7(0) |
| 15 | rs13329490 | 32408231 | 0.76 | 0.045 | 0.64 | 0.058 | CHRNA7(0) |
| 15 | rs904951 | 32418038 | 0.76 | 0.028 | 0.64 | 0.056 | CHRNA7(0) |
| 15 | rs1909884 | 32439298 | 0.76 | 0.034 | 0.64 | 0.063 | CHRNA7(0) |
| 15 | rs2611605 | 32441633 | 0.76 | 0.036 | 0.64 | 0.073 | CHRNA7(0) |
| 15 | rs7178176 | 32443813 | 0.76 | 0.039 | 0.64 | 0.066 | CHRNA7(0) |
| 15 | rs2926504 | 32509892 | 0.76 | 0.036 | 0.64 | 0.063 | CHRNA7(+48.66) |
| 15 | rs4072398 | 32510510 | 0.76 | 0.036 | 0.64 | 0.059 | CHRNA7(+49.27) |
| 15 | rs9672615 | 32511555 | 0.76 | 0.031 | 0.64 | 0.072 | CHRNA7(+50.33) |
| 15 | rs2946542 | 32513176 | 0.76 | 0.04 | 0.64 | 0.071 | CHRNA7(+51.95) |
| 15 | rs2946543 | 32513233 | 0.76 | 0.034 | 0.64 | 0.072 | CHRNA7(+52.01) |
| 15 | rs9672221 | 32514150 | 0.76 | 0.033 | 0.64 | 0.066 | CHRNA7(+52.93) |
| 15 | rs2611583 | 32514341 | 0.76 | 0.042 | 0.64 | 0.085 | CHRNA7(+53.12) |

**Table S3e.** Set of consecutive probes partially overlapping *CHRNA7* (15q13.3), associated with PC1 in the PLINK QFAM analysis of the CLDRC-RD subset.

^a^ Beta values are indicative of the direction of effect of the "CNV+" state (i.e. copy number other than 2) but are not adjusted for family-based structure of the dataset, as per PLINK QFAM output. ^b^ Physical distance (kb) from *CHRNA7* is indicated, along with orientation based on the direction of transcription ("-" = upstream of 5'-UTR, "+" = downstream of 3'-UTR).

S3f)

| Chr | SNP | Position (hg19) | P  (PC1) | P  (IQadjPC1) | Gene (distance)^a^ |
| --- | --- | --- | --- | --- | --- |
| 19 | rs8106213 | 20657781 | 1.6 x 10-3 | 3.5 x 10-3 | ZNF737(+63.01) |
| 19 | rs11669293 | 20663314 | 0.01 | 0.015 | ZNF737(+57.48) |
| 19 | rs2021399 | 20682055 | 6 x 10^-4^ | 2 x 10^-4^ | ZNF737(+38.74) |
| 19 | rs2545918 | 20691114 | 4 x 10^-4^ | 9 x 10^-4^ | ZNF737(+29.68) |
| 19 | rs4809060 | 20701612 | 4.5 x 10-3 | 9.6 x 10-3 | ZNF737(+19.19) |
| 19 | rs2545931 | 20704619 | 6.0 x 10-3 | 5.3 x 10-3 | ZNF737(+16.18) |
| 19 | rs4809062 | 20707568 | 0.026 | 0.013 | ZNF737(+13.23) |
| 19 | rs33948 | 20715228 | 9.5 x 10-3 | 7.3 x 10-3 | ZNF737(+5.57) |

**Table S3f.** Set of consecutive probes on 19p12 associated with PC1 and IQ-adjusted PC1 in the FamCNV analysis of the CLDRC-RD subset.

^a^ Physical distance (kb) from *ZNF737* is indicated, along with orientation based on the direction of transcription ("-" = upstream of 5'-UTR, "+" = downstream of 3'-UTR).

S3g)

| Chr | SNP | Position (hg19) | Beta  (PC1)^a^ | P-value (PC1) | Beta  (IQadjPC1)^a^ | P-value (IQadjPC1) | Gene (distance)^b^ |
| --- | --- | --- | --- | --- | --- | --- | --- |
| 19 | rs12610629 | 20626179 | 0.38 | 9.1 x 10-3 | 0.35 | 0.015 | ZNF737(+94.61) |
| 19 | rs10408291 | 20630360 | 0.37 | 0.014 | 0.34 | 0.01 | ZNF737(+90.43) |
| 19 | rs7254186 | 20631948 | 0.37 | 0.019 | 0.34 | 0.022 | ZNF737(+88.84) |
| 19 | rs10403597 | 20647550 | 0.37 | 8.8 x 10-3 | 0.34 | 0.012 | ZNF737(+73.24) |
| 19 | rs7251145 | 20656048 | 0.37 | 0.011 | 0.34 | 0.012 | ZNF737(+64.74) |
| 19 | rs8106213 | 20657781 | 0.37 | 0.015 | 0.34 | 0.017 | ZNF737(+63.01) |
| 19 | rs11669293 | 20663314 | 0.37 | 0.011 | 0.34 | 0.011 | ZNF737(+57.48) |
| 19 | rs2021399 | 20682055 | 0.37 | 0.01 | 0.34 | 0.011 | ZNF737(+38.74) |
| 19 | rs2545918 | 20691114 | 0.37 | 0.011 | 0.34 | 0.013 | ZNF737(+29.68) |
| 19 | rs4809060 | 20701612 | 0.37 | 0.013 | 0.34 | 0.013 | ZNF737(+19.19) |
| 19 | rs2545931 | 20704619 | 0.37 | 0.013 | 0.34 | 0.017 | ZNF737(+16.18) |
| 19 | rs4809062 | 20707568 | 0.37 | 0.014 | 0.34 | 0.018 | ZNF737(+13.23) |
| 19 | rs33948 | 20715228 | 0.39 | 0.013 | 0.36 | 0.013 | ZNF737(+5.57) |

**Table S3g.** Set of consecutive probes on 19p12 associated with PC1 and IQ-adjusted PC1 in the PLINK QFAM analysis of the CLDRC-RD subset.

^a^ Beta values are indicative of the direction of effect of the "CNV+" state (i.e. copy number other than 2) but are not adjusted for family-based structure of the dataset, as per PLINK QFAM output. ^b^ Physical distance (kb) from *ZNF737* is indicated, along with orientation based on the direction of transcription ("-" = upstream of 5'-UTR, "+" = downstream of 3'-UTR).

S3h)

| Candidate pathway | Pathway size  (nr of genes) | Overlaps | Empirical P | Corrected P |
| --- | --- | --- | --- | --- |
| axonal guidance ^a^ | 89 | 3 | 0.828 | 0.952 |
| neuronal migration ^b^ | 64 | 1 | 1 | 1 |
| steroids ^c^ | 333 | 5 | 0.999 | 0.999 |

S3i)

| Candidate pathway | Pathway size  (nr of genes) | Overlaps | Empirical P | Corrected P |
| --- | --- | --- | --- | --- |
| axonal guidance ^a^ | 89 | 2 | 1 | 1 |
| neuronal migration ^b^ | 64 | 0 | 1 | 1 |
| steroids ^c^ | 333 | 3 | 0.947 | 0.95 |

**Table S3.** Results of the pathway-based (INRICH) analysis of CNV calls detected in 67 RD cases in the CLDRC dataset. **h)** All CNVs detected (306); **i)** rare CNVs (84).

In this analysis, three global composite candidate pathways were tested, representing specific neurobiological hypotheses on the etiology of reading and language disabilities: axon guidance, neuronal migration and steroid sex hormone biology. ^a^ All the GO sets containing the term "axon guidance". ^b^ All the GO sets containing the term "neuron migration". ^c^ All the GO sets containing the terms "steroid", "androgen", "estrogen", "progesterone" and "testosterone".

S3a)


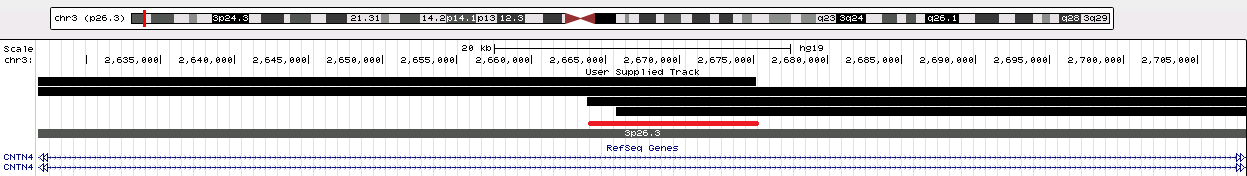


S3b)

**
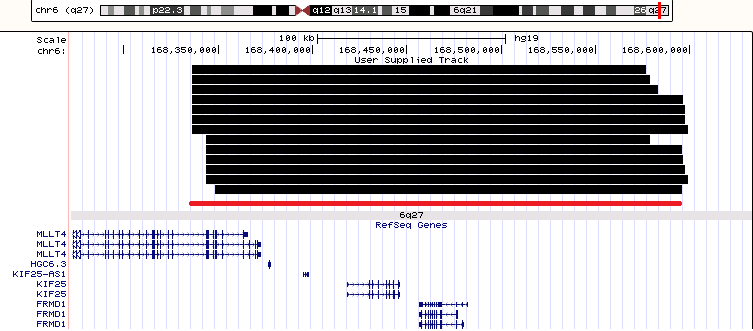
**

S3c)

**
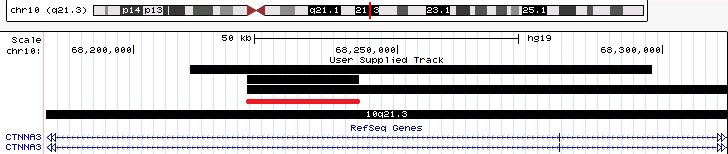
**

S3d)


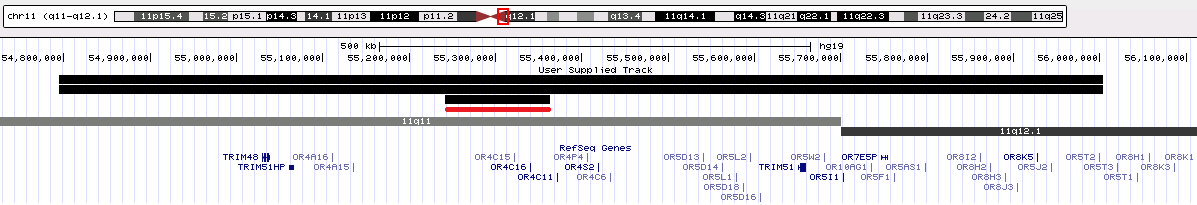


**Figure S3.** Regions of CNV overlap presenting the strongest associations with PC traits in the GWAS with CNV state (red line). **a)** chr3:2,663,757-2,675,189 (3p26.3); **b)** chr6:168,336,080-168,597,552 (6q27); **c)** chr10:68,221,549-68,242,672 (10q21.3); **d)** chr11:55,241,556-55,362,955 (11q11). Details on the association of these regions are reported in Table 4. Individual CNV calls are represented by black horizontal lines.
